# Modeling bioavailability to organs protected by biological barriers

**DOI:** 10.1186/2193-9616-1-8

**Published:** 2013-05-31

**Authors:** Nadia Quignot

**Affiliations:** Bioengineering Department, Chair of Mathematical Modeling for Systems Toxicology, Université de Technologie de Compiègne, Royallieu Research Center, Compiègne, 60200 France; LA-SER, Strategy and Decision Analytics, 10 place de la Catalogne, Paris, 75014 France

**Keywords:** Bioavailability, Biological barriers, Computational model, PBPK modeling, Pharmacokinetics

## Abstract

Computational pharmacokinetic (PK) modeling gives access to drug concentration *vs.* time profiles in target organs and allows better interpretation of clinical observations of therapeutic or toxic effects. Physiologically-based PK (PBPK) models in particular, based on mechanistic descriptions of the body anatomy and physiology, may also help to extrapolate *in vitro* or animal data to human.

Once in the systemic circulation, a chemical has access to the microvasculature of every organ or tissue. However, its penetration in the brain, retina, thymus, spinal cord, testis, placenta,… may be limited or even fully prevented by dynamic physiological blood-tissue barriers. Those barriers are both physical (involving tight junctions between adjacent cells) and biochemical (involving metabolizing enzymes and transporters).

On those cases, correct mechanistic characterization of the passage (or not) of molecules through the barrier can be crucial for improved PBPK modeling and prediction.

In parallel, attempts to understand and quantitatively characterize the processes involved in drug penetration of physiological barriers have led to the development of several *in vitro* experimental models. Data from such assays are very useful to calibrate PBPK models.

We review here those *in vitro* and computational models, highlighting the challenges and perspectives for *in vitro* and computational models to better assess drug availability to target tissues.

## Review

Computational tools permit to go beyond the frontiers of feasible experiments (McLanahan et al. 2012). They allow the generation and validation of mechanistic hypotheses and the number of results from simulations is virtually infinite. Pharmacokinetics (PK) models, and by extension physiologically-based PK (PBPK) models, aim at simulating quickly and at low cost the time course of the absorption, distribution, metabolism, and excretion (ADME) of a given drug in the body (Rostami-Hodjegan 2012). In doing so, they can provide predictions of a drug concentration in any organ or tissue (defined as a compartment) at any point in time. Access to drug concentration at the target cell level is important for understanding and predicting therapeutic or toxic effects; PBPK models are thus both important at the early stage of drug development and when assessing potential toxicity targets (Benjamin et al. 2010).

PBPK model equations and parameters characterize the various ADME processes and their interactions. Some of the absorption and distribution parameters may be estimated on the basis of tissue composition data and of the drug’s physicochemical properties via quantitative structure-properties models (Schmitt 2008). However, for estimating many of the PBPK parameters, *in vitro* experimental models have been developed and are essential (Cai et al. 2006).

There are many options when designing a PBPK model: the number of compartments is not limited, and many refinements are possible (Lee et al. 2009). Among them, the mechanistic description of passage through biological blood-tissue barriers appears very promising for both drug discovery and toxicity assessment. The challenges there are to characterize and predict permeation across the barriers, to design molecules which cross (or not) those barriers, and to have access to the effective chemical concentration in the target tissue.

In this review, we first recall the physiological basis for chemical distribution in tissues protected by biological barriers. We then describe the *in vitro* and computational tools to assess and predict barrier permeability. Finally, we provide an overview of challenges and perspectives in this area.

### Chemical distribution and physiological barriers

#### ADME processes

After entering the body, a drug follows an ADME scheme (Willmann et al. 2005; Leahy 2003). Absorption corresponds to the process by which the compound enters the systemic circulation. This process crucially varies according to the administration route, dose, and form. For example, the rate-limiting step for absorption following oral administration may be either the dissolution rate (function of drug physicochemical characteristics and the physiological environment), or the transport rate (permeability) across the intestinal epithelium (Lennernas 2007). Distribution involves mechanisms of drug dispersion and transport throughout the fluids and tissues of the body. Distribution can be limited by either perfusion (when the tissues present no barrier to diffusion), or permeability across vascular/tissue barrier or across cell membranes inside tissues (Geldof et al. 2008). Metabolism deals with the biotransformation of parent drugs into metabolites, by metabolic enzymes such as cytochromes P450, dehydrogenases, transferases… (Emoto et al. 2010; Yengi et al. 2007). Finally, excretion is the removal of the drugs (or their metabolites) from the body (Aimone 2005).

According to these ADME processes, the free concentration of a drug in a specific tissue usually depends on its plasma free concentration, the plasma/tissue barrier permeability, its tissue binding, cellular membrane permeability, and metabolic modifications by cellular enzymes. Physiological barriers may be encountered at all absorption, distribution, and excretion steps (Kitamura et al. 2008), from the skin and the intestinal barriers regulating absorption, to distribution at the level of several target tissues like brain or testis, and to excretion in kidney, intestine,… An exhaustive review of all those biological barriers is out of the scope of this work and we will focus on blood-tissue barriers limiting distribution, for which details are given next.

#### Blood-tissue barriers

**Figure 1:**
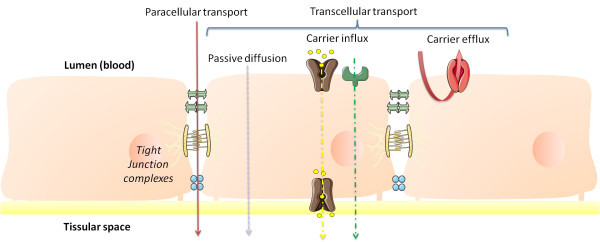
**Dynamics at the level of physiological barriers.** Epithelial and endothelial cell layers may form selectively permeable barriers, by which molecules pass either between the cells (paracellular route), or through the cells (transcellular route). The paracellular route is restricted by tight junction complexes, composed of communicating junctions, adherens junctions, and tight junctions. Influx mechanisms include carrier-mediated influx, receptor transcytosis, and absorptive-mediated transcytosis. The most known carrier efflux mechanism is mediated by P-glycoprotein.

The key role of blood-tissue barriers is to modulate and restrain permeability (Alexis et al. 2008). They are both physical and biochemical. Physical barriers consist in a layer of cells with closely associated membranes between adjacent cells. Membrane occlusion is mediated by protein complexes, like occludins and claudins for the tight junctions, cadherins for adherens junctions, and connexins for gap junctions. Biochemical barriers involve the metabolism of chemicals by metabolizing enzymes like cytochromes P450, the influx or efflux of chemicals by carrier protein like ATP-Binding cassette transporters such as P-glycoprotein (P-gp). A complex and dynamic multi-pathways process is involved in passage of molecules across biological compartments (Figure 1).

The transcellular pathway corresponds to the passage of molecules through cells (Cheung and Brace 2008). Passive diffusion through cell membranes is the preferential route for small lipophilic molecules (< 400 Da) (Levin 1980). Passage can also be carrier-mediation *via* facilitated diffusion or an active (or secondary active) process (Lockman et al. 2008). Facilitated diffusion corresponds to the transport of a molecule following its concentration gradient. Active processes involve energy to move a molecule against its electrochemical gradient. This energy can be provided 1) directly by the transporter itself which is able to hydrolyze ATP, or 2) secondary following the use of an ion concentration gradient. The compounds using those specific transporter systems are mostly hydrophilic.

The paracellular pathway permits the passage of small hydrophilic molecules through gaps existing between cells, despites cell junctions (Vandenbroucke et al. 2008). This kind of transport is considered to be passive (driven by diffusion). However, charge selectivity often occurs, notably for modulating the passage of molecules *via* pores between junctional proteins. Tight junctions between cells are most of the time organized in complexes: tight and adherens junctions for brain (Stamatovic et al. 2008); tight, adherens, and gap junctions for testes (Wong et al. 2004). Tight junctions fuse membranes of two adjacent cells. Adherens junctions physically connect the cytosquelettons of neighboring cells. Gap junctions connect two adjacent cells and form pores allowing small molecules to pass between the cytoplasm of neighboring cells (Li et al. 2012).

Another process involved in limitating the passage of molecules is efflux transport, by active transporters such as P-gp, which extrude back molecules after they have entered the cells (Edwards et al. 2005).

The presence of metabolizing enzymes in the layer of cells forming the barrier may also modulate chemical availability at the tissue level (Miksys and Tyndale 2009). For example, chemicals can eventually be metabolized into a more active compound, a fact exploited by pro-drugs (Cucullo et al. 2012).

The presence of barriers between blood and tissues is a challenge for PBPK modeling, because of their relative complexity. Fortunately, *in vitro* and computational methods intending to predict permeability have been developed and can be put to use.

### *In vitro* methods to evaluate chemical permeability through physiological barriers

*In vitro* models have been extensively used as alternative methodologies to *in vivo* models for evaluating the bioavailability at the level of organs protected by physiological barriers. Even if it is impossible to reproduce the complexity of *in vivo* systems, *in vitro* models can be designed with sufficient relevant features (Cucullo et al. 2011). An exhaustive review of all the available *in vitro* methods for assessing chemical permeability is out of the scope of this article (several reviews exist, see for example Sarmento et al. (2012)), and we chose to describe the main kinds of *in vitro* models, focusing on the endpoints measured and their relevance to PBPK model inputs.

#### Overview of the most commonly used *in vitro* models

According to the endpoint investigated, several *in vitro* models have been developed, ranging from the determination of partition coefficients to more mechanistic experiments (Table 1).

**Table 1:** ***In vitro*** **models for predicting barrier permeability/drug availability to protected organs**

***In vitro*** model		Principle	Examples	Advantages	Limitations
Tissue homogenates or slices		Measurement of partition coefficient by two main methods:	• Rat brain homogenates or slices (Friden et al. 2007)	• Good conservation of tissue organization and enzymatic capabilities	• Variability in preparations
		• Estimation of the fraction of unbound drug in the tissue by microdialysis of tissue homogenate against a drug-containing buffer solution			
		• Measure of the distribution of unbound drug in slices incubated in a drug-containing buffer			• No information on the dynamics of the process, an equilibrium value is obtained
Cell membrane preparations		Mechanistic characterization of specific target proteins (transporters in particular)	• Human cell membrane preparations (Miller et al. 2011)	• Simple, fast, cost-effective	• Presence of false positives
				• Focused assay system	• Lack of relevance (for example, transcriptional control cannot be taken into account)
				• Detailed mechanistic measurements feasible	• No metabolism
Cell cultures	Primary cells	Measurement of permeability endpoints:	• Rat primary sertoli cells (Siu et al. 2009)	• Large number of features similar to the *in vivo* phenotype	• Downregulation or altered expression of tight junctions, transporters, enzymes and receptors
		• Monolayer transepithelial electrical resistance		• Good ability to form efficient barrier *in vitro*	• Difficult to cultivate
	Cell lines	• Monolayer permeability to hydrophilic paracellular markers (lucifer yellow, sucrose, dextrans,…) between donor and receiver compartments	• Caco-2 cells (Inokuchi et al. 2009)	• When of human origin (most of time), better extrapolation than when of animal origin	• Less efficient in barrier straightness than primary cultures
				• Easy to use and cultivate	• Difficulty in obtaining the entire phenotype
				• Reproducible	• Genetic modifications: lack of relevance
	Co-cultures	Investigation of:	• Caco-2/HT29 co-culture (Antunes et al. 2013)	• Better representation of tissue heterogeneity	• Difficult to cultivate
		• Transport mechanisms			
		• Signaling pathways			
Immobilized artificial membranes		Measurement of passive permeation of compounds in a given environment (according to membrane composition)	• Immobilized artificial membranes with HPLC columns (Carrara et al. 2007)	• Ready-to-use	• Only account for passive permeability and do not assess potential active transport
				• Cost-effective	
				• Composition easily modifiable to mimic a tissue (addition of vesicles or liposomes in suspension, single phospholipid bilayers)	
			• Parallel artificial membrane permeability assay (Masungi et al. 2008)		

Cell-based assays consist of biological material from different levels of organization (from subcellular fractions to tissue fragments) and characterization (from well-described cellular transport processes to general information as used when determining partition coefficients). Tissue homogenates or slices can be used to estimate a drug’s partition coefficient between tissues and an extracellular medium (Friden et al. 2007). Cell membrane preparations are used to investigate particular pathways, transporters or receptors (Miller et al. 2011).

Some endothelial and epithelial cell cultures, when grown on permeable supports, spontaneously form monolayers and express functional junctions. Different levels of refinement are possible, from simple monolayers (Artursson et al. 2001; Trickler et al. 2010) to co-cultures (Antunes et al. 2013) to dynamic systems (Cucullo et al. 2011). These models allow dynamic permeability measurement through the monolayer. They also permit detailed characterization of biochemical mechanisms, such as receptor binding and uptake, or identification of relevant signaling pathways with mRNA and protein expression data (Seki et al. 2006).

Cell-based assays, however, are expensive and labor-intensive. In order to provide rapid, low-cost and automation-friendly tools to measure passive permeability, methods mimicking biological barriers with mixtures of lipids and organic solvents have been developed (Kv et al. 2008). For example, the immobilized artificial membranes (IAM) with HPLC (high-performance liquid chromatography) columns (Carrara et al. 2007) or the parallel artificial membrane permeability assay (PAMPA) give passive permeation estimates which correlate well with cell-based assays (Masungi et al. 2008).

Most of these tools can be automated for high throughput applications (Garberg et al. 1999). Because it is very difficult to develop a single *in vitro* system that can simulate the human *in vivo* setting, various *in vitro* assays are usually performed to investigate specific mechanisms (Abbott et al. 2008). Here, the key is to have a good knowledge of the barrier organization *in vivo*, both in terms of presence/absence of pathways and components, and in terms of their relative quantities.

#### Limitations of *in vitro* models and challenges for computational models

*In vitro* models have been optimized, becoming more and more complex in order to mimic as closely as possible biological barriers, and to permit investigation of several permeation mechanisms (Wuest et al. 2012; Hilgendorf et al. 2000).

Yet, *in vitro* systems remain quite simple and homogeneous, compared to the *in vivo* reality which involves several cell types, numerous processes, complex spatial structures, and much variability. It is difficult to obtain all the needed information in a given *in vitro* model, and computational tools allowing some representation of the complexity (*i.e.*, membrane transport systems, metabolism pathways, cell polarity, and extracellular composition) would be very useful.

A universal issue with *in vitro* systems is the relevance of their results to *in vivo* settings, and the conditions for validity of their extrapolation to *in vivo*. Physiological barriers are most of time complexes of junction proteins and involve both physical and biochemical mechanisms. It is rarely possible to assess the effects of all this components on resulting permeability in a unique stand-alone *in vitro* experiment. Firstly, it has been shown that junctions are tighter *in vivo* than *in vitro* (Garberg et al. 2005); the *in vitro* configuration, although being improved with tridimensional structures, still lacks relevance. Also, barrier protein functionality is under strong dependence of intercellular signals (like in the blood-testis barrier (Siu et al. 2009)); the *in vitro* conditions may not allow information to be shared between cells, as well as functional proteins to be expressed (Schug et al. 2013). Finally, the link between any *in vitro* model and *in vivo* conditions has to be clearly defined and quantified, ideally with well-justified mathematical descriptions. Spatial organization may also be illustrated computationally.

### Computational methods to describe and predict drug permeability through physiological barriers

PBPK models describe specific organs, tissues, and subcellular localizations as a set of pre-defined compartments linked together by the vascular system. In all those models, drug transport occurs at least via blood (Pilari and Huisinga 2010). Formally, they correspond to systems of differential equations for the concentrations or quantities of a given drug in each compartment. These compartments can be general (blood, “highly-perfused tissues”, “poorly perfused tissues”…) or very well described (Graf et al. 2012). In flow-limited models, the derivative for the quantity *X*_*T*_ of a drug *X* in tissue *T* is typically defined as:1

where *Q*_T_ is the blood flow rate, *C*_art_ the arterial blood concentration, *V*_*T*_ the tissue volume, and *P*_*T*_ the tissue-to-blood partition coefficient. The partition coefficient is still a meaningful parameter for an organ protected by a passive barrier, even if the model is quite simplistic. The rate of entry in the tissue will be over-estimated, however, thereby over-estimating tissue exposure.

A better approach for describing a permeability-limited transport (either transcellular, paracellular, or both) is to sub-compartmentalize the PBPK model: organs get usually sub-divided into three (vascular, extracellular and intracellular) compartments (Campbell 2009). Exchange rates between those compartments then fall back on linear processes, such as Fick’s law of diffusion (Kramer et al. 2009), or saturable transport if need is. A simpler sub-division into two sub-compartments will suffice to illustrate the model equations and introduce the needed parameters. The derivative for the quantity *X*_*TB*_ in the tissue vascular blood compartment of tissue *T* can be calculated as:2

where, in addition to the parameters and variables defined in eq. 1, *V*_*TB*_is the vascular blood volume in the tissue, *PS*_*T*_the apparent permeability-surface area product, *f*_*ub*_the unbound fraction of the compound in blood, *f*_*ut*_the unbound fraction of the compound in tissue, *X*_*TI*_the quantity of *X* in the interstitial and intracellular compartment and *V*_*TI*_the volume of that compartment (*V*_*TI*_= *V*_*T*_ – *V*_*TB*_).

For *X*_*TI*_, the corresponding differential equation is:3

To model active transport, a transport rate term *J* (nmol/min/mg protein) can be added to equation 3 and subtracted from equation 2 (for influx, the reverse if efflux is considered). *J* is usually based on conventional Michaelis-Menten kinetics:4

where *V*_*max*_ is the maximum transport capacity (nmol/min/mg protein), and *K*_*m*_ the half-saturation concentration. For efflux, the *TB* subscripts would be replaced by *TI*.

This leaves us with specific parameters to estimate. Such estimates can be obtained *in vitro*. Partition coefficients can be obtained either by model-based predictions according to the tissue composition (where the equation parameters are given by *in vitro* experiments on drug lipophilicity and plasma protein binding), or by *in vitro* direct determination of the drug's concentrations ratio in the buffer and tissue at steady-state (Poulin and Theil 2002b^2002^a; Rodgers et al. 2005; Rodgers and Rowland 2006). In cell monolayers or membranes *in vitro*, permeability can be evaluated using the equations for chemical flux, based on Fick’s first law for passive diffusion (Kramer et al. 2009). In that case, *in vitro* experiments allow a direct determination of an apparent permeability coefficient *P*_*app*_ (cm/s):5

where *dQ/dt* (mol/s) is the increase in the amount of drug/tracer molecule in the receiver compartment after a small time interval *dt* since time zero, *S* (cm^2^) the exchange surface, and *C*_*0*_ (mol/cm^3^) the initial drug/tracer molecule concentration in the donor chamber.

The apparent permeability-surface area product, *PS*_*T*_, is simply (Koda et al. 2007):6

Since junctions between adjacent cells restrict paracellular passage, a correction factor can be used when extrapolating from one barrier condition to another, for example when tight junctions are affected (Kondoh et al. 2012). Experimental permeability measurements may also correspond to the sum of transcellular, paracellular, or carrier-mediated passages (Amasheh et al. 2009). A specific permeability rate for paracellular passage can be assessed by using a tracer molecule which does not penetrate in cells (*e.g.*, lucifer yellow) (Inokuchi et al. 2009).

For active transport, determination of *V*_*max*_ and *K*_*m*_ follows simply the lines of Michaelis-Menten parameters estimation (Weiss and Kang 2002). When both active and passive transport are present, it is possible to fit a mixed model to the *in vitro* concentration time course data, at early times:7

The above parameters can also be estimated by fitting the whole PBPK model to *in vivo* PK data, but that is obviously not feasible in the absence of such data, as when *in vitro* to *in vivo* extrapolation is sought. The third option is to obtain those parameter estimates from computational "sub-models". Two main kinds of sub-models are used for that purpose: statistical models based on quantitative structure property relationship (QSPR), and mathematical models based on mechanistic description of biological processes.

#### Quantitative structure property relationship (QSPR)-based permeability models

One way to estimate the permeability of drugs through a given barrier is to link their chemical structures to the property of interest: permeability. The principle of QSPR models is that similar chemical structures should lead to similar properties (Sheridan et al. 2009). These are typical empirical statistical models, calibrated on a training dataset of chemicals of known structures and properties. Structures have to be somehow quantified through “descriptors” which enter the model as input variables, and the model gives a value for the property of interest (Neely et al. 2009). For a new drug, the calibrated model can therefore predict permeability, for example, simply on the basis of its chemical structure (provided that the new drug structure is not too far away from the structure of the drugs used in the training set).

The ability of drugs to traverse a tissue barrier is conditioned by tissue blood flow and several physicochemical properties: molecular size, lipophilicity, plasma protein binding, efflux pump affinity, molecular charge (Giaginis et al. 2009). QSPR modeling efforts for barrier-crossing have notably concentrated on passive permeability, for which enough data was available. As a further simplification, they concentrate on the prediction of partition coefficients (Chuman 2008), and mostly use molecular size and lipophilicity as descriptors. The impact of molecular size on paracellular permeability makes sense intuitively: the larger the molecule, the lower its ability to diffuse through the tight junctions of the tissue barrier. Lipophilicity property, often described by the octanol/water partition, Log P, influences the transcellular passage through lipid membranes.

Because the data necessary for predicting active or facilitated transport processes is currently insufficient, only a few QSPR models exist for those transport processes (Friedrichsen et al. 2001). Attempts to develop QSPR models for P-gp efflux have highlighted difficulties due to the broad specificity of this transporter (Gombar et al. 2004). Some QSPR models have also been developed for predicting enzymatic reactions like, for example, metabolic inactivation (Ekuase et al. 2011).

In spite of their usefulness for high-throughput screening of compounds on the basis of their physicochemical properties, QSPR models may lack predictive capacity (Chen et al. 2007). Mechanism-based models, which should lead to better predictions and on a time and space scale which would not be limited to the available information, are useful complements.

#### Mechanism-based permeability models

Another way to predict permeability is to describe its mechanisms according to biochemical and physical laws. A finer description of mechanisms, taking receptor uptake and transfer through membrane by carriers into account, can bring high value to PBPK models, in particular in the high dose range used in therapeutic applications (Tanaka et al. 1999).

There have been several efforts to develop computational ways to gain mechanistic insight in permeability processes. For example, Garmire et al. (2007) developed an *in silico* transwell device, incorporating both spatial (tight junctions) and functional (metabolizing enzymes) barrier features, for mimicking the *in vitro* passive transport properties through cell monolayers. Model predictions of passive transport were validated across *in vitro* data, and were reasonable approximations.

Another computational development is that of Dolghih and Jacobson (2013). They developed a computational approach of the blood–brain barrier combining two mechanism-based models: for passive permeation and for active efflux by P-gp. This model is a good illustration of the importance to combine permeability mechanisms for obtaining meaningful predictions.

### Perspectives for quantitative predictions of bioavailability to barriers-protected organs

#### Conceptualization

A well thought conceptualization is the key for developing a useful model. We have seen that there are several ways to build a PBPK model, from description of few global compartments to precise compartmentalization at the tissue or even at the cell levels. The same is true for parameters which can be estimated at whole tissue level or at a finer level.

One important thing in PBPK modeling is to have an integrated view of the whole body behavior. To model every detail is not feasible, and abstraction is essential to understand. Hence, an approach coupling general chemical behavior in the whole body and refinement through description at lower levels in tissues of special interest can give very useful insight about relevant mechanisms, while being predictive at a higher level.

The success of QSPR-based models depends both on the accuracy of algorithms and on the quality of input data. Because of the methodological and experimental variability present in published data, QSPR models may be poorly predictive. This highlights the need of a balance between i) the understanding of the reality of complex physiologic events to provide accurate systems information and ii) the simplicity required for computational modeling feasibility.

Different tools detailing each step of ADME processes are available and can be integrated into a coherent iterative approach (Honorio et al. 2012; Bois et al. 2010; Jamei et al. 2009). Yet again, the experimental evidence is the limit in developing mechanistic models. Thus, when designing a model, the balance has to be made between i) the available information and ii) the model complexity to meet the objectives.

#### Refinement

The increasing understanding of mechanisms intervening in chemical distribution allows the refinement of predictions about a chemical availability at the site of its effect, be it therapeutic or toxic. Another scale of complexity for chemical distribution may be to work at the level of cell organelles (Zheng et al. 2011).

As the area of systems biology is growing, investigating barrier formation mechanisms *via* signaling pathways can be of interest to predict behavior at a higher scale and earlier (Lee et al. 2010). Furthermore, besides the intrinsic functionality of physiological barriers, systems biology can also describe dynamical/homeostasis barrier mechanisms (Smallwood 2009).

#### Validation

As usual with computational models, usefulness and relevance have to be carefully assessed. Indeed, in the case of permeability models as in general, models of biological systems are strong on assumptions and weak on validation. A general framework for validation is provided by Sornette et al. (Sornette et al. 2007). They propose a formal iterative process, leading to the model rejection or progressive refinement and validation. In the particular case of computational models of biological systems, robust validation techniques against biological models have to be employed. Smallwood et al. (Smallwood et al. 2004) describe a modeling paradigm for developing a relevant predictive computational model of cellular interaction. In their example, the key is to understand the *in vitro* behavior. Cellular processes are stochastic, and the generation of distributions using Monte Carlo techniques appears to be the most relevant. Of course, the ideal would be to have a set of *in vitro* experiments and to apply a comparison metrics.

## Conclusions

Time-course of a drug concentration in organs protected by biological barriers can be obtained thanks to i) the time-course of concentration in blood and ii) the characterization of passage through those barriers. The first kind of information can be obtained from whole-body PBPK models, the second may come from several methods, *in vitro* experiments, QSPR models, or mechanism-based models defining a set of precise and highly specific parameters.

Both understanding of mechanisms and estimating model parameters in the field of barrier permeability can be done thanks to *in vitro* models. Of course, the complexity of the living systems and the imperfect predictive power of computational models mean that *in vitro* assays will be still used for a long time.

As long as experimental evidence come and scientific hypotheses are validated, the mathematical models for predicting passage across biological barriers will tend to be increasingly complex and realistic. They should thus lead to better extrapolation, from *in vitro* to *in vivo*, or from animal to human.
